# Atypical Presentation of Chronic Myelogenous Leukemia

**DOI:** 10.7759/cureus.1280

**Published:** 2017-05-26

**Authors:** Katelyn Mariko Updyke, Joan Morales-Lappot, Theodore Lee

**Affiliations:** 1 College of Medicine, University of Central Florida

**Keywords:** chronic myelogenous leukemia, leukemoid reaction, atrial fibrillation, pneumonia, myeloproliferative neoplasm, copd exacerbation

## Abstract

Chronic myelogenous leukemia (CML) is a clonal myeloproliferative disorder characterized by the presence of the Philadelphia chromosome, t(9;22), which is a constitutively active tyrosine kinase that causes excessive proliferation and differentiation of myeloid cells in the bone marrow. Most patients are either asymptomatic or present with fatigue, abdominal fullness, and splenomegaly. This is a case in which a 72-year-old Caucasian male’s initial presentation of CML was new-onset atrial fibrillation, chronic obstructive pulmonary disease (COPD) exacerbation, and pneumonia. The severity of his symptoms of atrial fibrillation and dyspnea complicated his stabilization, which delayed his diagnosis of CML and initiation of tyrosine kinase inhibitor for treatment. Unregulated proliferation of leukemic cells increases blood viscosity and results in aberrations in blood circulation that may result in atypical presenting symptoms in myeloproliferative disease. Thus, it is important to have a high clinical index of suspicion for CML in patients with leukocytosis and concurrent symptomatology that is unusual for leukemia.

## Introduction

Chronic myelogenous leukemia (CML) accounts for 15% of adult leukemias with the median age of diagnosis being 67 years [1]. CML is a clonal myeloproliferative disorder characterized by the presence of the Philadelphia chromosome, a balanced genetic translocation of chromosomes 22 and 9 known as the BCR-ABL fusion oncogene. The BCR-ABL fusion oncogene results in a constitutively active tyrosine kinase that causes excessive proliferation and differentiation of myeloid cells in the bone marrow [2]. Most patients are diagnosed during the chronic phase of CML, which is usually asymptomatic and diagnosed solely based on abnormal blood count, such as severe leukocytosis [3]. However, patients who do experience symptoms usually have fatigue, weight loss, early satiety, left upper quadrant pain with fullness and splenomegaly. However, there have been prior cases of atypical presentations of CML described. For example, there were two noted cases of recurrent painful priapism as initial presentation of CML [4]. Additionally, another patient with CML presented with syncope and myocardial infarction [5]. This is a case of a patient with new-onset CML presenting with *H. influenzae* pneumonia, severe emphysema exacerbation, new-onset atrial fibrillation with rapid ventricular rate, and leukocytosis. The patient’s atypical presentation and severe comorbidities complicated his stabilization, as he did not respond to multiple modes of treatment for his atrial fibrillation or breathing treatments for his dyspnea, thus delaying his diagnosis and initiation of treatment.

## Case presentation

The patient is a 72-year-old Caucasian male who presented to the emergency department with one-week history of productive cough with yellow-green sputum, general malaise, palpitations, and progressively worsening dyspnea. He had a past history of chronic obstructive pulmonary disease (COPD), and both pneumonia and a leukemoid reaction three months ago. On admission, he was found to have increased white blood cell count in the range of 70 x 10^9^/l. He was complaining of shortness of breath and palpitations, thus an electrocardiogram (EKG) and chest radiograph (CXR) were ordered. His EKG revealed an irregular rapid heart rate around 150-200 bpm, irregular rhythm and absent p-waves that were consistent with atrial fibrillation (a-fib) with rapid ventricular rate (RVR). Additionally, his CXR showed right lower lobe consolidation consistent with bacterial pneumonia. Sputum cultures were ordered, and it grew the bacteria *H. influenzae.* 

A physical examination revealed tachycardia with irregularly irregular rhythm, loud expiratory wheezes, moderate respiratory distress, scattered ecchymosis, but no splenomegaly. The patient's symptoms of shortness of breath and physical exam findings were consistent with severe COPD exacerbation. He was treated with intravenous steroids and nebulized bronchodilators with minimal improvement of his respiratory distress. Furthermore, his atrial fibrillation was equally challenging to manage; he was initially converted to sinus rhythm on amiodarone but had to be trialed with other treatments after his aspartate aminotransferase (AST) and alanine aminotransferase (ALT) were elevated on his liver function tests. He had an echocardiogram and cardiac stress test that showed reversible ischemia in the apical septum and an ejection fraction of 25%. He had a cardiac catheterization that was relatively unremarkable, and he was placed on sotalol to control his atrial fibrillation. On day 3 of his admission, his white count was elevated to 155.83 x 10^9^/l with 20% band neutrophils, 5% lymphocytes, 14% myelocytes, 2% promyelocytes and 1% blast cells. A peripheral blood smear revealed that he had an increased number of immature myeloid cells, metamyelocytes and myelocytes, basophils and eosinophils that were consistent with CML; however, a bone marrow biopsy was needed for confirmation. The patient’s bone marrow aspiration was postponed for several days until he was cleared by cardiology for the procedure. After he was cleared, his bone marrow biopsy revealed markedly hypercellular bone marrow (>80%) with granulocytic hyperplasia and positive BCR-ABL testing. The patient was subsequently started on dasatinib, a second generation tyrosine kinase inhibitor that exhibits activity against many clinically relevant BCR-ABL mutant forms [6]. The patient improved clinically after his white count began to trend down and stabilize in the range of 40 x 10^9^/l.

The patient was evaluated previously three months ago for leukocytosis (range in 40 x 10^9^/l) by a hematologist who did not determine any findings that were suggestive of leukemia on smear analysis and flow cytometry. The patient was lost to follow-up and did not have further testing.

## Discussion

The presented case illustrates the atypical clinical presentation of chronic myelogenous leukemia in this patient and the complexities in managing his symptoms. Upon initial presentation, the patient’s white blood cell count was elevated in the 70 x 10^9^/l range. At the time of admission, CML was a differential diagnosis but was not strongly considered due to the patient’s history of leukemoid reaction and his concurrent *H. influenzae* pneumonia. However, it is not uncommon for patients in an immunocompromised state, such as leukemia, to develop recurrent pneumonias [7]. Thus, in this patient the differential diagnoses should have been myeloproliferative disorder, leukemia, or sepsis. The patient’s recurring pneumonia exacerbated his underlying COPD leading to severe dyspnea and respiratory distress. There is a known risk of new-onset a-fib in patients with impaired pulmonary function, as hypercapnia and increased pulmonary artery systolic pressure have a direct relationship with stretching of the atria [8]. The patient had no history of cardiac disease or prior myocardial infarctions, thus his severe COPD and a-fib with RVR led to his cardiomyopathy and poor systolic function that delayed his bone marrow biopsy, diagnosis, and appropriate treatment.

## Conclusions

This case reiterates the importance of high clinical suspicion for myeloproliferative disorders in patients with elevated white blood counts, along with the importance for physicians to follow-up their patients in order to prevent significant complications of undiagnosed disease. Without careful tracking of his white blood cell count and further clinical investigation, the diagnosis of CML could have been missed.
